# A Multidomain Biomarker Score for Predicting Acute Kidney Injury and Renal Replacement Therapy Initiation in Critically Ill Adults

**DOI:** 10.3390/jcm15218351

**Published:** 2026-10-27

**Authors:** Semih Aydemir, Yavuz Kutay Gökçe, Doğukan Şenberber, Temel Kayan, Yavuz Katırcı, Dilber Kumral, Gamze Kılıçarslan, Onur Küçük, Fatih Segmen, Recep Dokuyucu

**Affiliations:** 1 Department of Anesthesiology and Reanimation, Yıldırım Beyazıt University Yenimahalle Training and Research Hospital, 06370 Ankara, Türkiye; dilberkumral1@gmail.com (D.K.); gamze88_ank@hotmail.com (G.K.); 2 Department of Internal Medicine, Yıldırım Beyazıt University Yenimahalle Training and Research Hospital, 06370 Ankara, Türkiye; ykgokce06@gmail.com; 3 Department of Anesthesiology and Reanimation, School of Medicine, Dokuz Eylül University, 35330 İzmir, Türkiye; dogukansenberber@gmail.com (D.Ş.); onur.kucuk@deu.edu.tr (O.K.); 4 Department of Intensive Care Unit, Ankara City Hospital, University of Health Sciences, 06800 Ankara, Türkiye; temel_kayan@hotmail.com; 5 Department of Emergency Medicine, Health Sciences University Gülhane Training and Research Hospital, 06010 Ankara, Türkiye; yavuz.katirci@sbu.edu.tr; 6 Department of Critical Care, Health Sciences University Ankara Bilkent City Hospital, 06800 Ankara, Türkiye; drsegmen@gmail.com; 7 Department of Physiology, Medical Specialization Training Center (TUSMER), 06420 Ankara, Türkiye; drecepfatih@gmail.com

**Keywords:** acute kidney injury, renal replacement therapy, critical illness, risk stratification, systemic inflammation, triglyceride–glucose index

## Abstract

**Background/Objectives**: We evaluated the Renal–Inflammatory–Nutritional–Metabolic Index (RIN-IM), a previously published 0–10-point composite biomarker score, for acute kidney injury (AKI) and renal replacement therapy (RRT) initiation in an independent intensive care unit (ICU) cohort. **Methods**: This retrospective study included 480 adults admitted to two ICUs between October 2023 and August 2026. RIN-IM was calculated within 24 h of admission from creatinine, albumin, systemic immune-inflammation index (platelet count × neutrophil count/lymphocyte count), pan-immune-inflammation value (neutrophil count × platelet count × monocyte count/lymphocyte count), and triglyceride–glucose index (triglyceride and admission glucose concentrations). Patients were classified by RIN-IM Score as low-risk (0–3 points), intermediate-risk (4–6 points), or high-risk (7–10 points). Among patients without AKI or acute RRT initiation at or before the 24-h landmark, subsequent incident AKI was the primary outcome and RRT initiation was the secondary renal outcome. Twenty-eight-day all-cause mortality was evaluated descriptively. Logistic regression and receiver operating characteristic analyses were performed. **Results**: AKI occurred in 189 patients (39.4%), RRT was initiated in 72 (15.0%), and 157 (32.7%) died within 28 days. In the low-, intermediate-, and high-risk groups, AKI frequencies were 16.0%, 40.0%, and 62.0%; RRT initiation frequencies were 3.3%, 12.2%, and 30.0%; and 28-day mortality was 18.0%, 32.2%, and 48.0%, respectively (all *p* < 0.001). Adjusted odds ratios per score point was 1.31 (95% confidence interval, 1.16–1.48) for AKI and 1.47 (1.25–1.73) for RRT initiation. Areas under the curve were 0.862 and 0.903, respectively, exceeding those of Acute Physiology and Chronic Health Evaluation II and Sequential Organ Failure Assessment. **Conclusions**: Higher RIN-IM scores were associated with AKI, RRT initiation, and higher observed 28-day mortality. The score may support early renal risk stratification, pending prospective assessment of calibration and clinical utility.

## 1. Introduction

Acute kidney injury (AKI) is a common complication of critical illness and is associated with prolonged intensive care unit (ICU) stay, renal replacement therapy (RRT), increased healthcare use, and adverse short- and long-term outcomes [1,2,3,4,5]. AKI is clinically heterogeneous: its onset and progression are influenced by pre-existing kidney reserve, hemodynamic instability, inflammation, infection, metabolic disturbances, and treatment-related exposures. Although the Kidney Disease: Improving Global Outcomes (KDIGO) criteria provide a standardized framework for diagnosing and staging AKI, they are primarily diagnostic criteria and depend on changes in serum creatinine and urine output that may become evident after kidney injury is already established [6,7].

Early renal risk stratification may help clinicians identify patients who warrant closer surveillance, review of nephrotoxic exposures, and timely optimization of hemodynamic and fluid management. General ICU severity scores, including Acute Physiology and Chronic Health Evaluation II (APACHE II) and Sequential Organ Failure Assessment (SOFA), are useful for describing global physiological derangement and prognosis [8,9,10,11]. However, they were not designed specifically for AKI or RRT prediction. A renal risk tool based on routinely available early laboratory data may therefore complement, rather than replace, established severity assessment.

The pathophysiology of AKI supports a multidomain approach. Serum creatinine reflects early renal functional impairment, whereas systemic immune-inflammatory activation may be captured by cell-count-derived indices such as the systemic immune-inflammation index (SII) and pan-immune-inflammation value (PIV) [12,13]. Higher PIV has been associated with adverse outcomes in patients with sepsis-associated AKI [14]. Hypoalbuminemia may reflect acute inflammation, capillary leak, catabolic stress, and reduced physiological reserve and has been consistently associated with AKI in hospitalized and critically ill populations [15,16,17]. In addition, the triglyceride–glucose (TyG) index integrates triglyceride and glucose concentrations and may capture metabolic stress; higher TyG values have been associated with kidney-related adverse outcomes in selected clinical settings [18,19,20,21].

The Renal–Inflammatory–Nutritional–Metabolic Index (RIN-IM) is a previously published score that combines serum creatinine, serum albumin, SII, PIV, and TyG using fixed, predefined thresholds [22,23]. It was originally derived to predict 28-day mortality in critically ill patients. Its components are available from routine early ICU laboratory testing and represent renal function, systemic immune-inflammatory activity, albumin-related changes during acute illness, and metabolic dysregulation. Whether the previously published RIN-IM Score, applied without modifying its original component thresholds or point assignments, can discriminate AKI and RRT initiation in an independent ICU population remains uncertain.

Therefore, this study evaluated the association and discriminatory performance of the previously published RIN-IM Score for AKI and RRT initiation in an independent multicenter cohort of critically ill adults. Twenty-eight-day all-cause mortality was assessed as a secondary clinical outcome. Discrimination for AKI and RRT initiation was compared with that of APACHE II and SOFA. The analysis was not designed as a formal external validation of the original mortality prediction model.

## 2. Materials and Methods

### 2.1. Study Design and Setting

This multicenter retrospective cohort study evaluated the prognostic performance of the previously published RIN-IM Score for renal outcomes in critically ill adults. The original RIN-IM algorithm was developed from routinely available early ICU laboratory measurements to predict 28-day mortality and incorporates serum creatinine, serum albumin, the systemic immune-inflammation index (SII), the pan-immune-inflammation value (PIV), and the triglyceride–glucose (TyG) index [22]. No patient included in the present cohort contributed to the derivation cohort of the original RIN-IM study.

This retrospective study included adult patients admitted to the ICUs of Ankara Bilkent City Hospital and Ankara Yıldırım Beyazıt University Yenimahalle Training and Research Hospital between October 2023 and August 2026. These dates define the patient admission period. The study protocol was approved by the Ankara Yıldırım Beyazıt University Yenimahalle Training and Research Hospital Non-Interventional Scientific Research Ethics Committee (Approval No. E-2025-9; approval date: 16 October 2025). Research-specific medical-record review, data extraction, and statistical analyses were undertaken after ethics approval. Renal outcomes were assessed after the 24-h index time until ICU discharge or death, and all-cause mortality was assessed within 28 days of ICU admission. All procedures conformed to the Declaration of Helsinki and applicable institutional regulations.

Patients were eligible if they were aged 18 years or older, remained in the ICU for at least 48 h, and had complete clinical and laboratory data required for calculation of the RIN-IM Score and ascertainment of renal outcomes. Patients receiving chronic maintenance hemodialysis or peritoneal dialysis, those with a previous kidney transplant, and those with missing, inconsistent, or insufficient data required for RIN-IM Score calculation or outcome ascertainment were excluded. The required data comprised serum creatinine, serum albumin, platelet, neutrophil, lymphocyte, and monocyte counts, triglycerides, and admission glucose. For the incident renal-outcome analyses, patients were additionally required to have no KDIGO-defined AKI and no acute RRT initiation at or before the 24-h index time. All available pre-index creatinine measurements and urine-output records were reviewed to identify AKI present at hospital admission or developing before the landmark. Patients without a reliable preadmission creatinine measurement or urine-output documentation sufficient for AKI ascertainment were excluded from these analyses. The complete-data requirement applied to the primary complete-case analysis. For sensitivity analyses, otherwise eligible patients with incomplete predictor data were also considered if their kidney status at the index time and the relevant renal outcome could be reliably established.

### 2.2. Data Collection

All demographic, clinical, and laboratory variables were obtained from the hospital’s electronic medical record system. Demographic and clinical data included age, sex, comorbidities such as hypertension, diabetes mellitus, heart failure, chronic pulmonary disease, and malignancy, as well as smoking status and the primary indication for ICU admission. Laboratory measurements comprised serum creatinine, blood urea nitrogen, estimated glomerular filtration rate, C-reactive protein, complete blood count used to derive the neutrophil-to-lymphocyte ratio, serum albumin, total protein, admission glucose, and lipid parameters including triglycerides. Laboratory measurements used to calculate the RIN-IM Score were the first eligible measurements recorded during routine clinical care within 24 h of ICU admission. These values were subsequently extracted retrospectively from the electronic medical records for the present study. In addition, disease-severity indices, including the Acute Physiology and Chronic Health Evaluation II (APACHE II) and Sequential Organ Failure Assessment (SOFA) scores, were extracted to enable comparative evaluation of predictive performance. Baseline serum creatinine was defined as the most recent outpatient measurement obtained during clinical stability within 7–365 days before hospital admission. Measurements associated with a documented acute illness were not accepted as baseline values. Patients without a reliable preadmission measurement were excluded from the incident renal-outcome analyses. The creatinine measurement used to calculate RIN-IM was distinguished from the historical baseline value used for AKI ascertainment.

AKI was diagnosed and staged using both serial serum creatinine measurements and hourly urine output according to KDIGO criteria [6,24,25]. Diagnostic thresholds were a creatinine rise of ≥0.3 mg/dL over 48 h, a ≥1.5-fold increase relative to baseline developing within 7 days, or urine output < 0.5 mL/kg/h sustained for at least 6 h. Severity was assigned using the highest stage reached by either criterion. Initiation of acute RRT for AKI was classified as stage 3.

Incident AKI was defined as the first fulfillment of KDIGO criteria after the 24-h index time in patients who had not met these criteria at or before the landmark. The secondary renal outcome was the first initiation of acute RRT after the index time. Renal outcomes were followed until ICU discharge or death. Twenty-eight-day all-cause mortality was measured from ICU admission and retained as a descriptive secondary clinical outcome [6,24,25].

### 2.3. RIN-IM Score Calculation

The RIN-IM Score was calculated according to the previously published algorithm, without re-estimation of component weights or modification of the original thresholds in the present validation cohort [22]. The score integrates five routinely available early ICU laboratory measures representing renal dysfunction, systemic immune-inflammatory activation, albumin-related alterations during acute illness, and metabolic stress: serum creatinine, serum albumin, systemic immune-inflammation index (SII), pan-immune-inflammation value (PIV), and the triglyceride–glucose (TyG) index.

The index time was defined as 24 h after ICU admission. For each RIN-IM component, the first eligible laboratory measurement collected during the initial 24 h and available by the index time was used. Measurements obtained after the index time were not used to calculate the score. Renal outcomes were assessed strictly after this landmark. SII was calculated as platelet count × neutrophil count/lymphocyte count, and PIV as neutrophil count × platelet count × monocyte count/lymphocyte count; all blood-cell counts were expressed as ×10^9^/L. The TyG index was calculated as ln[[triglycerides (mg/dL) × admission glucose (mg/dL)]/2]. Because fasting status could not be confirmed uniformly in critically ill patients, TyG was interpreted as a marker of early metabolic dysregulation rather than a direct measure of chronic insulin resistance.

Each component was assigned 0, 1, or 2 points using the originally reported cutoffs: serum creatinine < 1.50, 1.50–1.99, and ≥2.00 mg/dL; SII < 1000, 1000–2999, and ≥3000; PIV < 500, 500–1199, and ≥1200; serum albumin ≥ 3.50, 2.50–3.49, and <2.50 g/dL; and TyG index < 9.00, 9.00–9.49, and ≥9.50, respectively. Component points were summed without additional weighting, producing a total RIN-IM Score from 0 to 10.

For descriptive analyses, patients were classified using the predefined score categories of low-risk (0–3 points), intermediate-risk (4–6 points), and high-risk (7–10 points). These categories and the individual component thresholds were retained unchanged from the original derivation study; they were not recalibrated or selected according to AKI, RRT, or mortality outcomes in the present cohort. Estimated glomerular filtration rate, C-reactive protein, and neutrophil-to-lymphocyte ratio were assessed separately when available but were not included in the RIN-IM Score calculation (Table 1).

For exploratory sensitivity analyses, a creatinine-free score was calculated by subtracting the creatinine component’s assigned points from the total RIN-IM Score. This derived score comprised albumin, SII, PIV, and TyG and ranged from 0 to 8 points. The original thresholds and point assignments of the retained components were unchanged. The original RIN-IM risk categories were not applied to this exploratory score.

### 2.4. Statistical Analysis

All statistical analyses were performed using IBM SPSS Statistics version 27 (IBM Corp., Armonk, NY, USA) and R software (version 4.3.0; R Foundation for Statistical Computing, Vienna, Austria). Continuous variables were assessed for normality using the Shapiro–Wilk or Kolmogorov–Smirnov test and summarized as mean ± standard deviation for approximately normally distributed data or median with interquartile range (IQR) otherwise. Categorical variables were presented as counts and percentages. Comparisons across the three predefined RIN-IM Score categories were performed using one-way analysis of variance for approximately normally distributed continuous variables and the Kruskal–Wallis test otherwise. Categorical variables were compared using Pearson’s chi-square test or Fisher’s exact test, as appropriate. Separate binary logistic regression models were fitted to examine the associations of the RIN-IM Score with any AKI and RRT initiation. The score was entered as a continuous variable, with odds ratios and 95% confidence intervals reported per 1-point increment. Both primary multivariable models used the same clinical adjustment set: age (per 10-year increment), sex, chronic kidney disease, sepsis at ICU admission, and study center. Age and sex were included to account for demographic differences, chronic kidney disease for pre-existing renal disease, sepsis for sepsis-related renal risk, and study center for potential differences in case mix and clinical practice. These models evaluated covariate-adjusted associations of the RIN-IM Score with renal outcomes. APACHE II and SOFA were evaluated as comparator scores in receiver operating characteristic analyses and were not included as adjustment covariates because of their overlapping renal information. Individual RIN-IM components were not entered separately into the primary models.

Receiver operating characteristic (ROC) analyses compared the discrimination of the RIN-IM, APACHE II, and SOFA scores for AKI and RRT initiation. Additional analyses addressing the potential influence of shared creatinine information were performed in the same primary complete-case cohort. Discrimination for subsequent incident AKI was compared among the original RIN-IM Score, serum creatinine alone, and the creatinine-free score. The creatinine comparator was the same early measurement used to calculate the RIN-IM Score, rather than the historical reference value used for KDIGO ascertainment. For all receiver operating characteristic analyses, areas under the curve and their 95% confidence intervals were reported, and paired comparisons were performed using the DeLong test.

To assess whether the non-creatinine components provided additional predictive information beyond the early creatinine measurement, two nested logistic regression models were compared within the same primary complete-case cohort. The reference model included serum creatinine and the same clinical adjustment covariates used in the primary analyses; the extended model additionally included the creatinine-free score. Both models were fitted to identical patients and compared using a likelihood ratio test. The adjusted odds ratio per 1-point increment in the creatinine-free score was reported with its 95% confidence interval.

The amount and joint patterns of missing or unusable data were summarized before complete-case exclusion, overall and by study center. Missing values and unresolved inconsistencies were reported separately. Patients included in the primary analysis were compared with those excluded because of insufficient data using available demographic and clinical characteristics. Variable-specific observed denominators and standardized differences were reported.

As a sensitivity analysis, missing predictor values were handled using multiple imputation by chained equations, generating 50 imputed datasets under a missing-at-random assumption. This analysis included otherwise eligible patients whose index-time kidney status and relevant renal outcome were ascertainable. The imputation models included the underlying laboratory measurements, clinical adjustment variables, study center, and the observed renal outcome. Continuous variables were imputed using predictive mean matching and categorical variables using appropriate regression models. SII, PIV, TyG, and the RIN-IM Score were recalculated within each completed dataset using their original definitions and thresholds. Imputation diagnostics were examined for convergence and plausibility. The primary adjustment set was retained, and regression coefficients and variances were pooled using Rubin’s rules.

Center-specific analyses reported sample sizes, outcome frequencies, and AUCs with 95% confidence intervals for the RIN-IM, APACHE II, and SOFA scores for AKI and RRT initiation. The original RIN-IM component thresholds and point assignments were retained. These analyses were considered exploratory.

Exploratory calibration and prediction-error analyses were conducted for incident AKI and RRT initiation in the primary complete-case cohort. For each outcome, separate logistic regression models containing the RIN-IM, APACHE II, or SOFA score as the sole continuous predictor were used to obtain predicted probabilities. These models were distinct from the covariate-adjusted association models. Calibration was summarized using calibration-in-the-large and calibration slope. Overall prediction error was quantified using the Brier score, with lower values indicating lower prediction error. The constant-risk reference Brier score was calculated as π(1 − π), where π was the observed event proportion.

Twenty-eight-day all-cause mortality was evaluated as a descriptive secondary clinical outcome and summarized as counts and percentages overall and across the predefined RIN-IM Score categories, with unadjusted between-group comparisons. Regression and receiver operating characteristic analyses were restricted to the renal outcomes. A two-sided *p*-value < 0.05 was considered statistically significant.

## 3. Results

**Figure 1:**
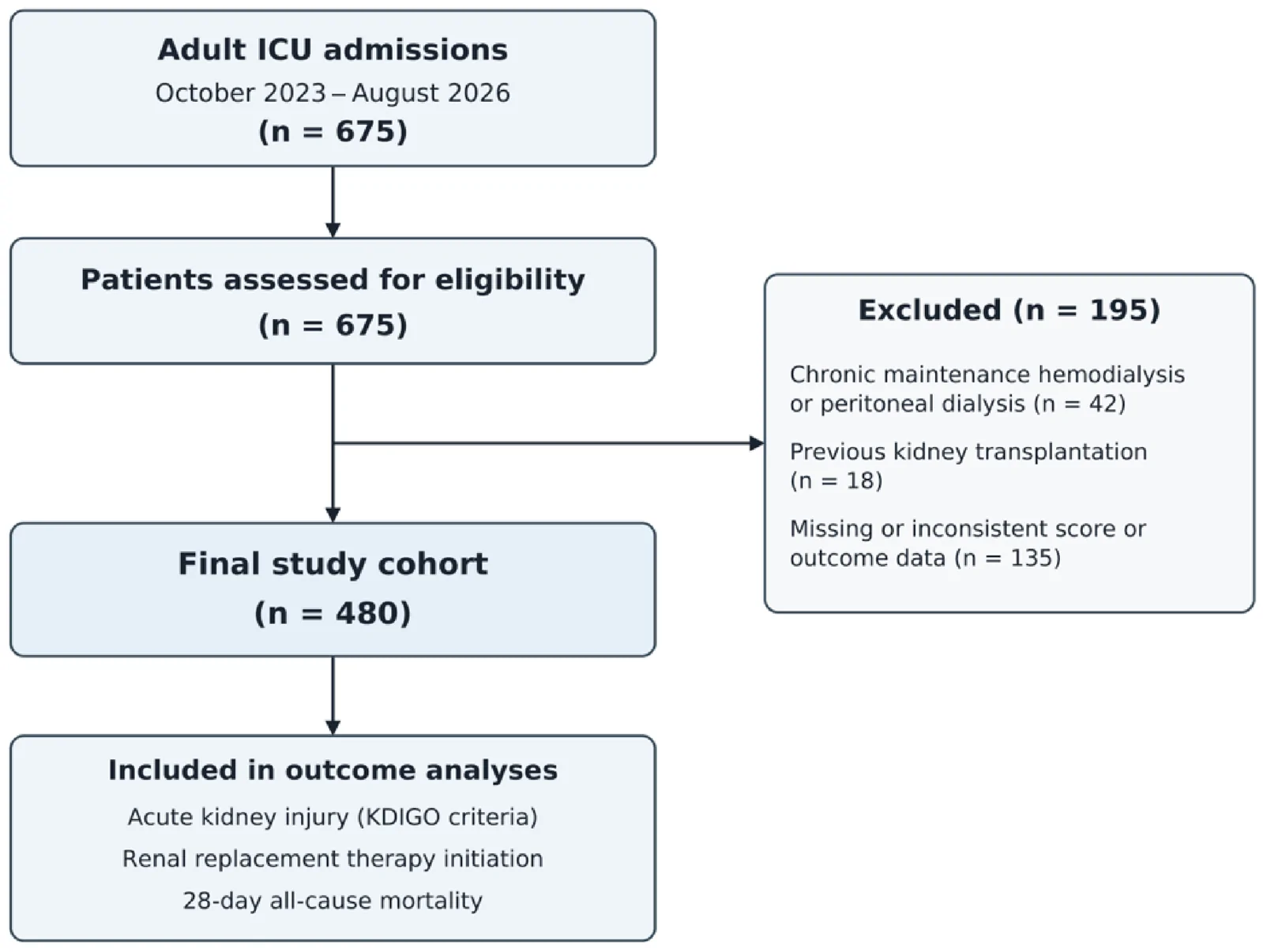
Flow diagram of patient selection and outcome assessment.

During the study period, a total of 675 adult patients admitted to the ICU were screened for eligibility, of whom 480 fulfilled the inclusion criteria and were included in the final analysis after exclusions. Sample size justification was based on the primary outcome of KDIGO-defined acute kidney injury (AKI). Assuming an AKI incidence of approximately 40% in critically ill patients, we anticipated around 190 AKI events in a cohort of 480 patients. Using the commonly recommended rule of at least 10–15 events per variable for logistic regression and planning to include approximately 10 predictors in the multivariable model, this number of events was considered sufficient to ensure stable coefficient estimates for the primary analysis. The expected incidence of renal replacement therapy (RRT) in this population was lower, around 10–20%, corresponding to roughly 70 RRT events in 480 patients. Therefore, analyses involving RRT were predefined as secondary and exploratory, and more parsimonious multivariable models with a reduced number of predictors were used to maintain an acceptable ratio of events per variable (Figure 1).

Baseline characteristics according to RIN-IM Score category are presented in Table 2. Patients in the high-risk category were older than those in the intermediate- and low-risk categories, whereas sex distribution and body mass index were comparable across groups. The prevalences of hypertension, diabetes mellitus, chronic kidney disease, and heart failure differed across RIN-IM Score categories, with the highest values in the high-risk category (all *p* < 0.001). Illness severity also differed across groups, with progressively higher APACHE II and SOFA scores in the low-, intermediate-, and high-risk categories (both *p* < 0.001). As expected from the prespecified score composition, higher RIN-IM categories were characterized by higher serum creatinine, SII, PIV, triglyceride, admission glucose, and TyG values, together with lower serum albumin concentrations (all *p* < 0.001) (Table 2).

Incident acute kidney injury occurred in 189 of the 480 patients (39.4%) during follow-up after the 24-h index assessment. The frequencies were 16.0%, 40.0%, and 62.0% in the low-, intermediate-, and high-risk RIN-IM categories, respectively (*p* < 0.001). The distribution of the highest KDIGO stage reached during follow-up differed across score categories (overall *p* < 0.001; Table 3). Overall, 85 patients reached stage 3, including 72 who received acute RRT for AKI and 13 who met stage 3 creatinine or urine-output criteria without receiving RRT (Table 3).

Among the 72 patients receiving acute RRT for AKI, the primary indications were refractory fluid overload with pulmonary edema in 27 (37.5%), refractory hyperkalemia in 19 (26.4%), severe metabolic acidosis in 19 (26.4%), and uremic complications in 7 (9.7%). One primary indication was assigned per patient. Twenty-eight-day mortality was 18.0%, 32.2%, and 48.0% in the low-, intermediate-, and high-risk categories, respectively (*p* < 0.001; Table 4).

In univariable analysis, each 1-point increase in the RIN-IM Score was associated with higher odds of both any AKI and RRT initiation. After adjustment for age, sex, chronic kidney disease, sepsis at ICU admission, and study center, the RIN-IM Score remained associated with AKI (adjusted OR per point, 1.31; 95% CI, 1.16–1.48; *p* < 0.001) and RRT initiation (adjusted OR per point, 1.47; 95% CI, 1.25–1.73; *p* < 0.001). Chronic kidney disease was also associated with both outcomes in adjusted analyses (Table 5).

**Figure 2:**
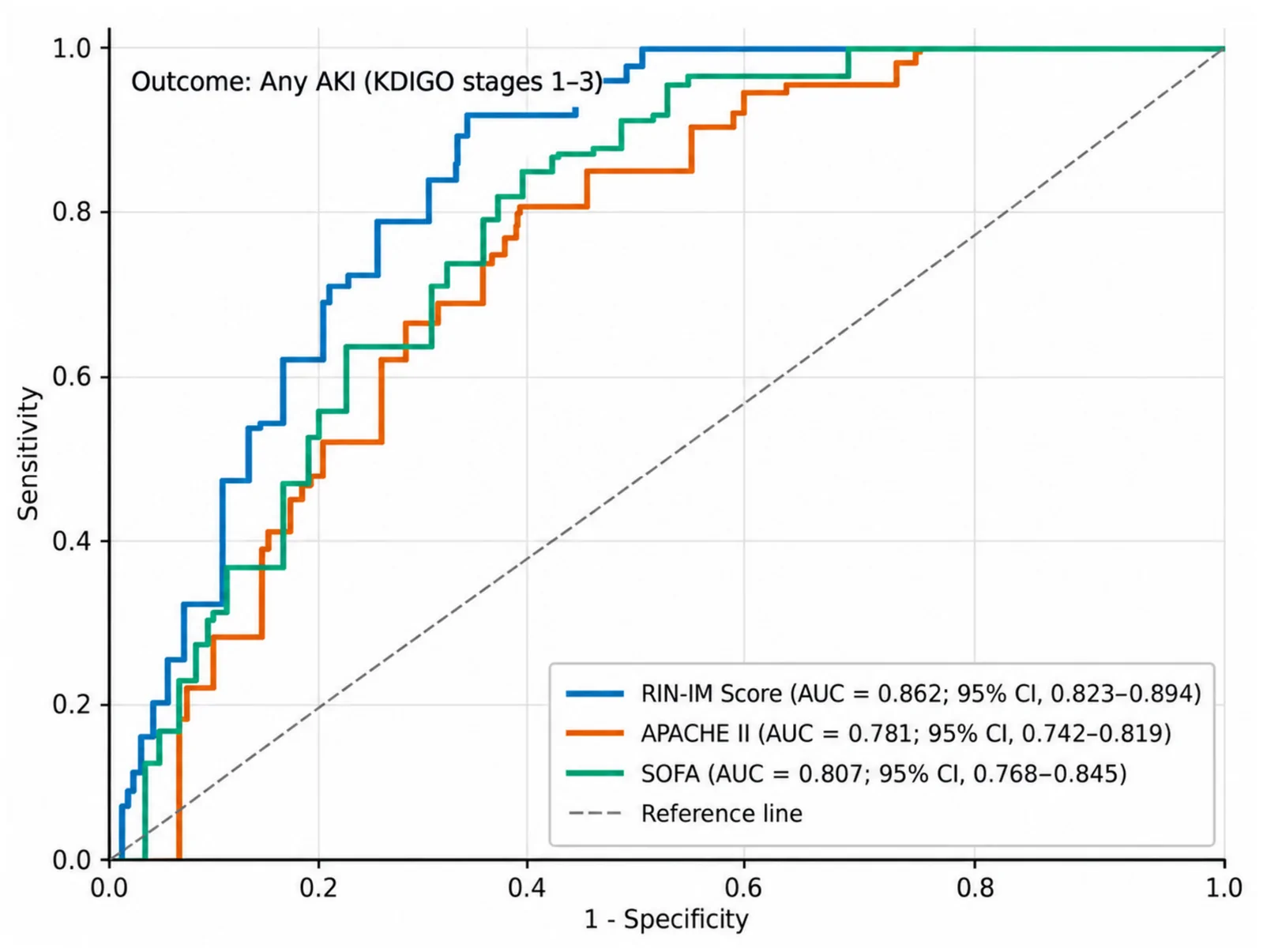
ROC curves of the RIN-IM Score, APACHE II, and SOFA for predicting any acute kidney injury.

**Figure 3:**
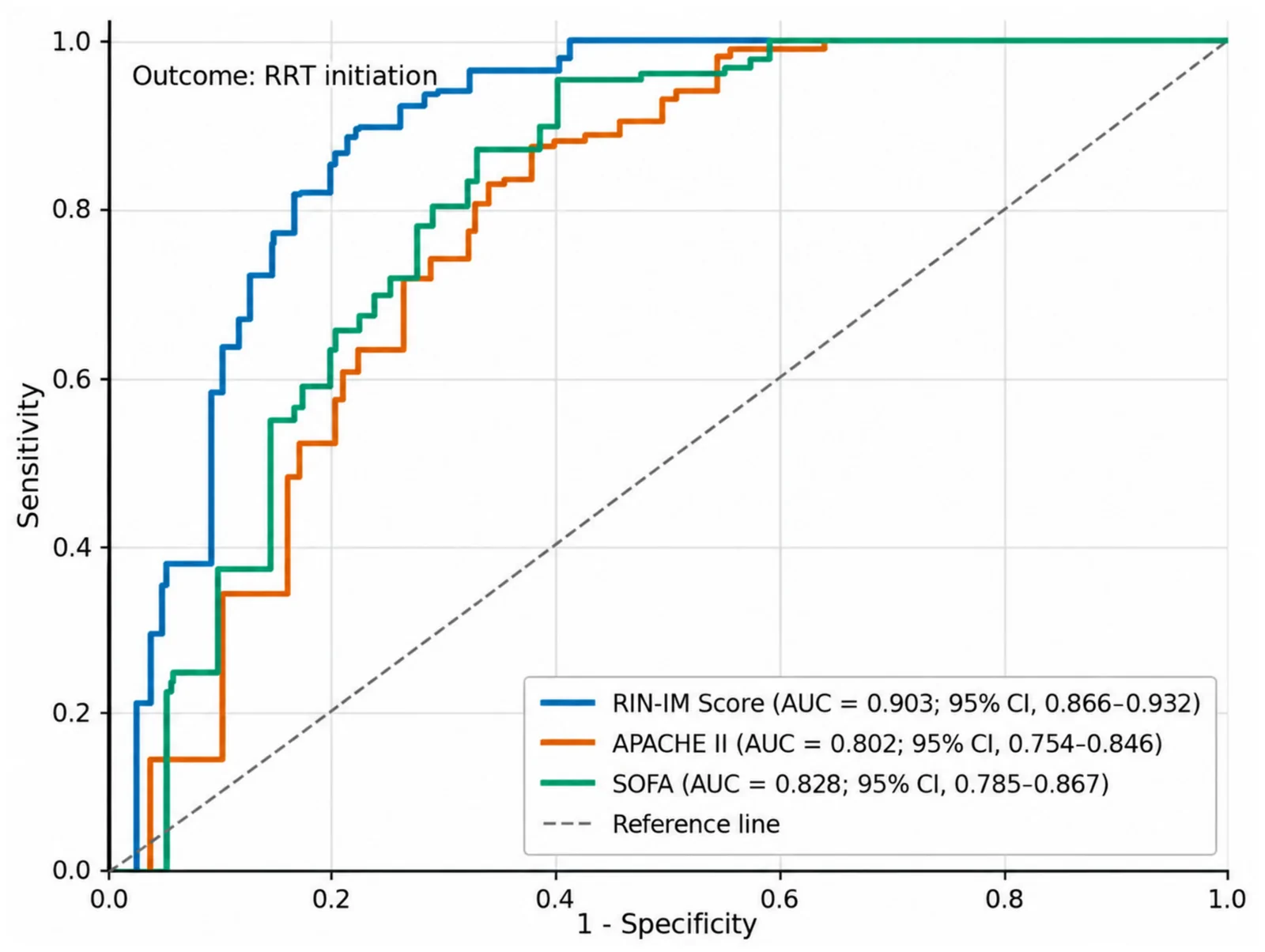
ROC curves of the RIN-IM Score, APACHE II, and SOFA for predicting renal replacement therapy initiation.

The RIN-IM Score showed good discrimination for AKI and RRT initiation (Table 6; Figure 2 and Figure 3). Exploratory cutoffs of ≥5 points for AKI and ≥6 points for RRT initiation were selected by maximizing the Youden index in the same cohort.

For the prediction of any AKI, the AUC of the RIN-IM Score was higher than those of APACHE II (difference in AUC, 0.081; 95% CI, 0.038–0.124; *p* < 0.001) and SOFA (difference in AUC, 0.055; 95% CI, 0.015–0.095; *p* = 0.007). Similar findings were observed for RRT initiation, for which the RIN-IM Score showed higher AUCs than APACHE II (difference, 0.101; 95% CI, 0.047–0.155; *p* < 0.001) and SOFA (difference, 0.075; 95% CI, 0.025–0.125; *p* = 0.003). The differences between APACHE II and SOFA were not statistically significant for either outcome (Table 7).

Sensitivity analyses comparing the original RIN-IM Score with serum creatinine alone and the creatinine-free score in patients without AKI or acute RRT initiation at the index time are presented in Supplementary Table S1.

Variable-level missingness and joint patterns of missing or unusable data are summarized in Supplementary Table S2. Available demographic and clinical characteristics of patients included in the primary analysis and those excluded because of insufficient data are compared in Supplementary Table S3.

Multiple-imputation sensitivity analyses and center-specific sample sizes, event frequencies, and discrimination estimates for AKI and RRT initiation are presented in Supplementary Table S4.

Exploratory calibration and prediction-error measures for the outcome-specific score-based models are presented in Supplementary Table S5.

## 4. Discussion

In this independent multicenter ICU cohort, higher RIN-IM Score categories were associated with higher observed frequencies of AKI, severe AKI, and RRT initiation, as well as higher 28-day mortality. In adjusted analyses, each 1-point increment in the RIN-IM Score was associated with higher odds of both AKI and RRT initiation. The score also demonstrated greater discrimination than APACHE II and SOFA for both renal outcomes. Taken together, these findings indicate that a fixed multidomain biomarker score may provide clinically relevant renal risk stratification when applied early during ICU admission.

AKI remains a frequent and consequential complication of critical illness. In the AKI-EPI study, Hoste et al. reported AKI in approximately one-half of critically ill patients, with important implications for mortality and resource utilization [4]. Birkelo et al. further emphasized that AKI and acute kidney disease are associated with both short- and long-term adverse outcomes in ICU populations [2]. The 39.4% incidence of AKI in the present cohort is consistent with this substantial burden, although direct comparisons across cohorts require caution because case mix, baseline kidney function, duration of observation, and the use of serum creatinine and urine-output criteria may differ.

The RIN-IM Score was originally specified as a five-component score comprising serum creatinine, serum albumin, SII, PIV, and the TyG index [22]. No component thresholds or weights were re-estimated in the present analysis. This is an important feature because it permits assessment of the score as a fixed tool rather than development of a new, cohort-specific model. Its biological rationale is also relevant to renal risk in critical illness: early renal dysfunction, systemic immune-inflammatory activation, hypoalbuminemia during acute illness, and metabolic stress may coexist and amplify susceptibility to kidney injury.

The inflammatory component of the score deserves particular consideration. SII and PIV incorporate circulating neutrophils, lymphocytes, platelets, and, for PIV, monocytes; they may therefore reflect a broader immune-inflammatory state than a single biomarker. In sepsis-associated AKI, Zhou and Hu reported an association between higher PIV and mortality, supporting the prognostic relevance of dysregulated immune-inflammatory activity in renal injury [14]. In the present cohort, increasing SII and PIV values were observed across ascending RIN-IM categories, as expected from the prespecified scoring algorithm. These findings should be interpreted as evidence of biological coherence rather than proof that either index independently causes AKI. Nevertheless, combining two complementary inflammation-based indices may capture aspects of immune activation and microvascular injury that are not directly represented by conventional global severity scores.

Hypoalbuminemia may identify patients with impaired physiological reserve, systemic inflammation, capillary leak, and acute catabolism. Hansrivijit et al. found that lower serum albumin was associated with an increased risk of AKI across hospitalized populations, while Chen et al. reported that nutrition-related composite indices were associated with AKI and mortality in critically ill adults [15,16]. Albumin is not a specific nutritional marker during acute illness; however, its inclusion in RIN-IM may add information about the host response that is not conveyed by creatinine or leukocyte-derived indices alone. In this study, serum albumin concentrations decreased across increasing RIN-IM categories, consistent with this multidimensional risk profile.

The TyG component provides a metabolic dimension to the score. Previous studies have associated higher TyG values with kidney-related and cardiometabolic outcomes [18,19,20,26]. Little et al. reviewed the mechanisms linking impaired insulin signalling with systemic metabolic and vascular dysfunction [26]. This provides biological context for considering metabolic abnormalities in a multidomain score, but does not establish that admission-based TyG accurately measures insulin resistance in critically ill patients. In this study, fasting status was not consistently documented, and glucose and triglyceride concentrations could reflect acute illness, treatment, and nutritional exposure. Accordingly, TyG was interpreted as an indicator of early metabolic dysregulation; its association with renal outcomes should not be taken as evidence that chronic insulin resistance was directly measured.

APACHE II and SOFA remain valuable tools for characterizing overall physiological derangement in ICU patients, although they were not designed specifically to quantify renal risk [11,27]. In this cohort, the RIN-IM Score had higher standalone AUCs for AKI and RRT initiation than these comparator scores. These comparisons describe relative discrimination and do not establish incremental predictive value from adding RIN-IM to an existing clinical prediction model. Similarly, a covariate-adjusted association does not by itself demonstrate improved predictive performance. The present findings therefore support further evaluation of RIN-IM for renal risk stratification, while its additional value beyond established clinical predictors remains to be determined.

The categorical findings may have practical implications. Patients classified as high-risk had a substantially greater occurrence of any AKI, severe AKI, and RRT initiation. If confirmed prospectively, the score could help identify patients for closer monitoring of renal function and urine output, review of nephrotoxic exposure, optimization of hemodynamic and fluid management, and timely nephrology input. Such use should remain supportive rather than directive. In particular, the RIN-IM Score should not determine the initiation of RRT, which must continue to be based on the full clinical context and established indications for kidney support [6].

This study has several strengths. It evaluated a previously specified score in a separate contemporary multicenter cohort without patient overlap with the original derivation study [22]. The score was calculated from routinely available early laboratory data and was assessed against clinically important renal outcomes, including AKI severity and RRT initiation. In addition, comparison with APACHE II and SOFA, together with formal DeLong testing, provides a transparent description of relative discrimination. The inclusion of 28-day mortality as a secondary outcome also demonstrates that higher renal-risk categories identified a clinically more vulnerable population.

## 5. Limitations

Several limitations should be acknowledged. First, the retrospective design is susceptible to residual confounding, incomplete documentation, and variation in clinical practice between centers. RRT initiation is influenced not only by kidney dysfunction but also by hemodynamic status, fluid balance, clinician judgment, and local practice patterns. Second, the score was calculated from a single early measurement window; serial changes in its components and their potential association with evolving AKI risk were not examined. Third, serum creatinine is both a component of RIN-IM and a component of the KDIGO AKI definition. Even when renal outcomes are assessed after the index measurement period, partial incorporation bias remains possible and may contribute to the observed association with AKI. The 24-h landmark design restricts interpretation to patients who remained free of AKI and acute RRT through this time and does not address earlier renal events. Exclusion of patients without adequate historical creatinine or urine-output documentation may introduce selection bias. Temporal separation of predictors and outcomes does not eliminate the overlap arising from the inclusion of creatinine in both RIN-IM and AKI ascertainment.

Fourth, the RIN-IM Score was originally developed for 28-day mortality [22]. Accordingly, the present analysis is not a formal external validation of the original mortality prediction model; rather, it evaluates the association and discrimination of the fixed score for different renal outcomes in an independent cohort. Furthermore, 28-day mortality was assessed using descriptive statistics and unadjusted group comparisons, without mortality-specific multivariable regression or receiver operating characteristic analyses. Therefore, the observed mortality differences across score categories do not establish an association independent of the measured clinical covariates or comparative mortality-prediction performance relative to APACHE II and SOFA. Fifth, calibration and prediction error were assessed exploratorily using outcome-specific score-based probability models (Supplementary Table S5). These cohort-derived probability models require external validation. Calibration curves were not presented, and clinical net benefit was not evaluated using decision curve analysis. Therefore, these exploratory assessments do not establish clinical utility or support routine implementation. A high AUC does not by itself establish that a score provides accurate absolute risk estimates or improves clinical decision-making. The incremental predictive value of the full RIN-IM Score was not evaluated by comparing otherwise identical clinical models with and without the score. Consequently, its added value beyond established clinical predictors or severity scores remains uncertain. Finally, the number of RRT events was modest, limiting the precision of adjusted estimates and subgroup analyses.

Prospective multicenter studies should evaluate RIN-IM in patients with clearly defined baseline kidney status and prespecified incident AKI outcomes. Such studies should report calibration measures, assess performance across major ICU subgroups, compare the score with established AKI-specific tools and damage biomarkers, and determine whether serial score assessment adds value beyond the initial measurement. Only after this evidence is available should RIN-IM-guided monitoring or kidney-protective care pathways be tested for their effect on patient-centered outcomes.

## 6. Conclusions

In conclusion, in this independent multicenter ICU cohort, higher RIN-IM Score categories were associated with greater occurrence and severity of AKI, more frequent RRT initiation, and higher 28-day mortality. The fixed score showed good discrimination for AKI and RRT and had higher AUCs than APACHE II and SOFA for these renal outcomes. Because its components are routinely available during early ICU care, RIN-IM warrants further evaluation for renal risk stratification, including assessment of its incremental value beyond established clinical predictors. However, it should not replace clinical judgment or determine RRT initiation. Prospective external validation of the renal-outcome probability models, including assessment of calibration and clinical utility, is required before routine implementation.

## Figures and Tables

**Table 1:** Components, point assignments, and risk categories of the RIN-IM Score.

**A. Score components and point assignments**			
**Score component**	**0 points**	**1 point**	**2 points**
Serum creatinine, mg/dL	<1.50	1.50–1.99	≥2.00
Systemic immune-inflammation index (SII)	<1000	1000–2999	≥3000
Pan-immune-inflammation value (PIV)	<500	500–1199	≥1200
Serum albumin, g/dL	≥3.50	2.50–3.49	<2.50
Triglyceride–glucose (TyG) index	<9.00	9.00–9.49	≥9.50
**B. Total RIN-IM Score, points**		**Risk category**	
0–3		Low risk	
4–6		Intermediate risk	
7–10		High risk	

SII was calculated as platelet count × neutrophil count/lymphocyte count, and PIV as neutrophil count × platelet count × monocyte count/lymphocyte count. All blood-cell counts were expressed as ×10^9^/L. The TyG index was calculated as ln[[triglycerides (mg/dL) × admission glucose (mg/dL)]/2]. Each component contributes 0–2 points; the inflammatory domain contributes up to 4 points because it includes both SII and PIV. The component thresholds, point assignments, and risk categories were retained from the original derivation study without modification [22].

**Table 2:** Baseline demographic, clinical, and laboratory characteristics of the study population stratified by RIN-IM risk categories.

Variable	Low RIN—IM (*n* = 150)	Intermediate RIN—IM (*n* = 180)	High RIN—IM (*n* = 150)	*p*-Value
**Demographic and clinical characteristics**				
Age, years	59.8 ± 14.6	64.7 ± 13.8	69.5 ± 12.6	<0.001
Male sex, *n* (%)	88 (58.7)	104 (57.8)	94 (62.7)	0.620
Body mass index, kg/m^2^	27.3 ± 4.8	27.0 ± 5.0	26.6 ± 4.9	0.410
Hypertension, *n* (%)	61 (40.7)	94 (52.2)	103 (68.7)	<0.001
Diabetes mellitus, *n* (%)	38 (25.3)	72 (40.0)	81 (54.0)	<0.001
Chronic kidney disease, *n* (%)	9 (6.0)	27 (15.0)	54 (36.0)	<0.001
Heart failure, *n* (%)	21 (14.0)	42 (23.3)	56 (37.3)	<0.001
Chronic lung disease, *n* (%)	18 (12.0)	31 (17.2)	38 (25.3)	0.006
Active malignancy, *n* (%)	9 (6.0)	18 (10.0)	21 (14.0)	0.048
**Illness severity at ICU admission**				
APACHE II score	14.8 ± 5.7	19.6 ± 6.4	24.7 ± 7.1	<0.001
SOFA score	4.2 ± 1.8	6.1 ± 2.4	8.4 ± 3.1	<0.001
**Laboratory variables within the first 24 h**				
Blood urea nitrogen, mg/dL	22.7 ± 8.1	32.6 ± 10.4	46.9 ± 14.8	<0.001
Estimated glomerular filtration rate, mL/min/1.73 m^2^	84.1 ± 15.6	67.2 ± 18.1	43.8 ± 17.6	<0.001
Serum creatinine, mg/dL	1.02 ± 0.25	1.42 ± 0.41	2.19 ± 0.75	<0.001
Serum albumin, g/dL	3.72 ± 0.31	3.12 ± 0.39	2.43 ± 0.39	<0.001
Systemic immune-inflammation index	720 (520–910)	1680 (1160–2320)	3760 (2840–5140)	<0.001
Pan-immune-inflammation value	330 (240–440)	720 (530–980)	1510 (1190–2040)	<0.001
Triglycerides, mg/dL	133 ± 39	171 ± 54	217 ± 66	<0.001
Admission glucose, mg/dL	122 ± 26	151 ± 42	184 ± 52	<0.001
TyG index	8.91 ± 0.26	9.35 ± 0.22	9.83 ± 0.27	<0.001
RIN—IM Score, points	1.8 ± 0.8	5.0 ± 0.8	8.0 ± 0.7	— *

Data are presented as mean ± standard deviation, median (interquartile range), or *n* (%), as appropriate. Continuous variables were compared using one-way analysis of variance or the Kruskal–Wallis test, and categorical variables using the chi-square test or Fisher’s exact test, as appropriate. Laboratory variables were obtained from the first eligible measurement within 24 h of ICU admission. SII, systemic immune-inflammation index; PIV, pan-immune-inflammation value; TyG, triglyceride–glucose; ICU, intensive care unit. * No *p*-value is reported for the RIN—IM Score because patients were classified into groups using this score: low-risk, 0–3 points; intermediate-risk, 4–6 points; and high-risk, 7–10 points. A comparison of the score across these groups would therefore reflect the grouping criteria rather than an independent between-group difference.

**Table 3:** Incidence and severity of acute kidney injury after the index assessment according to RIN-IM Score category.

**A. Distribution of KDIGO AKI severity**				
**KDIGO AKI category**	**Low RIN—IM** **(*n* = 150)**	**Intermediate** **RIN—IM (*n* = 180)**	**High RIN—IM** **(*n* = 150)**	***p*** **-value**
No AKI	126 (84.0)	108 (60.0)	57 (38.0)	
Stage 1 AKI	18 (12.0)	36 (20.0)	33 (22.0)	
Stage 2 AKI	5 (3.3)	22 (12.2)	28 (18.7)	
Stage 3 AKI	6 (4.0)	26 (14.4)	53 (35.3)	
Of stage 3: receiving acute RRT for AKI	5 (3.3)	22 (12.2)	45 (30.0)	
Of stage 3: not receiving RRT	1 (0.7)	4 (2.2)	8 (5.3)	
Overall comparison of the four-category distribution				<0.001
**B. Comparison of any AKI versus no AKI**				
**Outcome**	**Low RIN—IM** **(*n* = 150)**	**Intermediate** **RIN—IM (*n* = 180)**	**High RIN—IM** **(*n* = 150)**	***p*** **-value**
Any AKI (KDIGO stages 1–3), *n* (%)	24 (16.0)	72 (40.0)	93 (62.0)	<0.001

Values are presented as *n* (%), with percentages calculated using the total number of patients in each RIN-IM Score category. AKI was classified according to the Kidney Disease: Improving Global Outcomes (KDIGO) criteria. The *p*-value in Panel A compares the four-category KDIGO severity distribution across score groups. The *p*-value in Panel B compares the binary outcome of any AKI versus no AKI across the same groups.

**Table 4:** Renal replacement therapy requirement and 28-day mortality rates according to RIN-IM risk groups.

Outcome	Low RIN-IM (*n* = 150)	Intermediate RIN-IM (*n* = 180)	High RIN-IM (*n* = 150)	*p*-Value
**Renal outcome**				
Acute RRT initiated for AKI after the 24-h index time, *n* (%)	5 (3.3)	22 (12.2)	45 (30.0)	<0.001
**Secondary clinical outcome**				
All-cause death within 28 days, *n* (%)	27 (18.0)	58 (32.2)	72 (48.0)	<0.001

Percentages are calculated using the number of patients in each RIN-IM Score category as the denominator. RRT, renal replacement therapy. RRT initiation was assessed after the index clinical and laboratory assessment. Acute RRT initiation was assessed after the 24-h index time and before ICU discharge or death. Mortality refers to all-cause death within 28 days of ICU admission.

**Table 5:** Univariable and multivariable logistic regression analyses for AKI and RRT.

Variable	Univariable OR (95% CI)	*p*-Value	Multivariable OR (95% CI)	*p*-Value
**A. Any AKI (KDIGO stages 1–3)**				
RIN-IM Score, per 1-point increase	1.48 (1.33–1.64)	<0.001	1.31 (1.16–1.48)	<0.001
Age, per 10-year increase	1.19 (1.06–1.34)	0.004	1.07 (0.93–1.23)	0.350
Male sex	1.12 (0.79–1.60)	0.530	1.07 (0.72–1.60)	0.740
Chronic kidney disease	3.12 (1.93–5.04)	<0.001	1.82 (1.06–3.12)	0.030
Sepsis at ICU admission	2.14 (1.49–3.08)	<0.001	1.57 (1.04–2.38)	0.032
Study center 2 versus center 1	1.09 (0.75–1.57)	0.660	1.03 (0.70–1.54)	0.870
**B. RRT initiation**				
RIN-IM Score, per 1-point increase	1.72 (1.52–1.95)	<0.001	1.47 (1.25–1.73)	<0.001
Age, per 10-year increase	1.21 (1.01–1.45)	0.040	1.10 (0.88–1.38)	0.410
Male sex	1.08 (0.65–1.79)	0.770	1.01 (0.58–1.77)	0.970
Chronic kidney disease	4.35 (2.45–7.71)	<0.001	2.29 (1.17–4.47)	0.016
Sepsis at ICU admission	2.37 (1.40–4.00)	0.001	1.60 (0.87–2.94)	0.130
Study center 2 versus center 1	1.16 (0.67–2.00)	0.590	1.10 (0.59–2.04)	0.770

Separate binary logistic regression models were fitted for any AKI and RRT initiation. Each multivariable model included the RIN-IM Score, age, sex, chronic kidney disease, sepsis at ICU admission, and study center. Serum creatinine, albumin, SII, PIV, and TyG were not entered separately because they are components of the RIN-IM Score. APACHE II and SOFA were evaluated separately as comparator scores and were not included in these regression models. OR, odds ratio; CI, confidence interval; AKI, acute kidney injury; RRT, renal replacement therapy; ICU, intensive care unit.

**Table 6:** Discriminatory performance of the RIN-IM Score, APACHE II, and SOFA for acute kidney injury and renal replacement therapy.

Score	Outcome	AUC	95% CI	Exploratory Cutoff	Sensitivity, %	Specificity, %	Youden Index	*p*-Value
RIN-IM	Any AKI	0.862	0.823–0.894	≥5	81.3	78.4	0.596	<0.001
APACHE II	Any AKI	0.781	0.742–0.819	≥19	71.5	70.2	0.417	<0.001
SOFA	Any AKI	0.807	0.768–0.845	≥6	74.8	72.6	0.474	<0.001
RIN-IM	RRT initiation	0.903	0.866–0.932	≥6	84.2	81.0	0.652	<0.001
APACHE II	RRT initiation	0.802	0.754–0.846	≥22	73.6	72.1	0.457	<0.001
SOFA	RRT initiation	0.828	0.785–0.867	≥7	76.4	74.3	0.507	<0.001

Exploratory cutoffs were selected by maximizing the Youden index in the same cohort used to assess performance. *p* values refer to tests of the AUC against the null value of 0.50. AUC, area under the receiver operating characteristic curve; CI, confidence interval; AKI, acute kidney injury; RRT, renal replacement therapy.

**Table 7:** Pairwise comparison of receiver operating characteristic curve areas using the DeLong test.

Outcome	Comparison	Difference in AUC (95% CI)	Z-Value	*p*-Value
**Any AKI**	RIN-IM versus APACHE II	0.081 (0.038–0.124)	3.68	<0.001
	RIN-IM versus SOFA	0.055 (0.015–0.095)	2.69	0.007
	SOFA versus APACHE II	0.026 (−0.012–0.064)	1.34	0.180
**RRT initiation**	RIN-IM versus APACHE II	0.101 (0.047–0.155)	3.67	<0.001
	RIN-IM versus SOFA	0.075 (0.025–0.125)	2.94	0.003
	SOFA versus APACHE II	0.026 (−0.020–0.072)	1.11	0.267

Positive AUC differences indicate a higher AUC for the first-listed score. Pairwise comparisons were performed using the DeLong test for correlated receiver operating characteristic curves. AUC, area under the receiver operating characteristic curve; CI, confidence interval; AKI, acute kidney injury; RRT, renal replacement therapy.

## Data Availability

Data is available upon request to the corresponding author.

## Supplementary Materials

The following supporting information can be downloaded at https://www.mdpi.com/article/10.3390/jcm15218351/s1. Table S1: Sensitivity analyses assessing the contribution of creatinine and the non-creatinine components of the RIN-IM Score to incident AKI prediction. Table S2: Amount and patterns of missing or unusable data before complete-case exclusion. Table S3: Comparison of patients included in the primary analysis and those excluded because of insufficient data. Table S4: Missing-data sensitivity analyses and center-specific discrimination for renal outcomes. Table S5: Exploratory calibration and prediction-error measures for outcome-specific score-based models.

## Abbreviations

AKI	Acute Kidney Injury
APACHE II	Acute Physiology and Chronic Health Evaluation II
AUC	Area Under the Curve
BMI	Body Mass Index
BUN	Blood Urea Nitrogen
CI	Confidence Interval
CRP	C-reactive Protein
eGFR	Estimated Glomerular Filtration Rate
ICU	Intensive Care Unit
KDIGO	Kidney Disease: Improving Global Outcomes
NLR	Neutrophil-to-Lymphocyte Ratio
OR	Odds Ratio
RIN-IM	Renal–Inflammatory–Nutritional–Metabolic Index
SII	Systemic immune-inflammation index
PIV	Pan-immune-inflammation value
ROC	Receiver Operating Characteristic
RRT	Renal Replacement Therapy
SOFA	Sequential Organ Failure Assessment
TyG	Triglyceride–Glucose Index
