# Supplementary material for: A Multidomain Biomarker Score for Predicting Acute Kidney Injury and Renal Replacement Therapy Initiation in Critically Ill Adults
Source: J Clin Med. 2026 Oct 27;15(21):8351. doi: 10.3390/jcm15218351 (PMC13763669; doi:10.3390/jcm15218351)
Supplement: Supplementary file 1 [file jcm-15-08351-s001.zip › jcm-4584343-supplementary.pdf]

Supplementary Table S1. Sensitivity analyses assessing the contribution of creatinine and the non-creatinine components of the RIN-IM Score to incident AKI prediction.

A. Analysis population

**Characteristic**	**Value**
Patients assumed to be free of AKI and acute RRT initiation at the index time, n	480
Subsequent incident AKI, n (%)	189 (39.4)
No subsequent AKI, n (%)	291 (60.6)

B. Discrimination for subsequent incident AKI

**Predictor**	**AUC**	**95% CI**
Original RIN-IM Score, 0–10 points	0.771	0.722–0.819
Serum creatinine alone, mg/dL	0.759	0.711–0.807
Creatinine-free score, 0–8 points	0.764	0.716–0.812

C. Paired comparisons of discrimination

**Comparison**	**Difference in AUC**	**95% CI for difference**	**DeLong p-value**
Original RIN-IM Score versus serum creatinine alone	0.012	−0.027 to 0.051	0.551
Original RIN-IM Score versus creatinine-free score	0.007	−0.005 to 0.019	0.274
Creatinine-free score versus serum creatinine alone	0.005	−0.041 to 0.051	0.832

D. Additional contribution of the creatinine-free score beyond serum creatinine and clinical covariates

**Analysis**	**Estimate**	**95% CI**	**p-value**
Creatinine-free score, per 1-point increment, in the extended model	Adjusted OR: 1.31	1.14–1.52	<0.001
Extended model versus reference model	Likelihood ratio χ^2: 14.53; df = 1	—	<0.001

AKI, acute kidney injury; AUC, area under the receiver operating characteristic curve; CI, confidence interval; df, degrees of freedom; ICU, intensive care unit; OR, odds ratio; PIV, pan-immune-inflammation value; RIN-IM, Renal–Inflammatory–Nutritional–Metabolic Index; RRT, renal replacement therapy; SII, systemic immune-inflammation index; TyG, triglyceride–glucose index. The analyses used the same 480-patient primary complete-case cohort described in the main manuscript. Patients were required to be free of KDIGO-defined AKI and acute RRT initiation at or before the index time, defined as 24 hours after ICU admission. Subsequent incident AKI occurred in 189 patients, whereas 291 patients remained free of AKI during follow-up. Comparisons of the original RIN-IM Score, serum creatinine alone, and the creatinine-free score used the same patients and outcome definition. The creatinine-free score was calculated by subtracting the creatinine component's assigned points from the original RIN-IM Score. It comprised albumin, SII, PIV, and TyG, retained their original thresholds and point assignments, and ranged from 0 to 8 points. The original RIN-IM risk categories were not applied to this exploratory score. The serum creatinine comparator was the early measurement used for score calculation, rather than the historical reference creatinine used for KDIGO ascertainment. AUC confidence intervals and paired AUC comparisons were calculated using the DeLong method. Positive AUC differences indicate higher discrimination for the first-listed predictor. Comparisons were exploratory, and two-sided p-values were not adjusted for multiple comparisons. The reference logistic regression model included early serum creatinine as a continuous variable, age per 10-year increment, sex, chronic kidney disease, sepsis at ICU admission, and study center. The extended model additionally included the creatinine-free score. Both models were fitted to the same analysis population. The adjusted odds ratio for the creatinine-free score was reported per 1-point increment, with its 95% confidence interval and p-value calculated using the Wald method. Model fit was compared using a likelihood ratio test with one degree of freedom.

Supplementary Table S2. Amount and patterns of missing or unusable data before complete-case exclusion.

A. Demographic and clinical characteristics

**Variable**	**Missing, n**	**Unusable, n**	**Unavailable overall, n (%) (N = 615)**	**Center 1 n (%) (N = 329)**	**Center 2, n (%) (N = 286)**
Age	0	0	0 (0.0)	0 (0.0)	0 (0.0)
Sex	0	0	0 (0.0)	0 (0.0)	0 (0.0)
Chronic kidney disease	0	0	0 (0.0)	0 (0.0)	0 (0.0)
Sepsis at ICU admission	0	0	0 (0.0)	0 (0.0)	0 (0.0)
Study center	0	0	0 (0.0)	0 (0.0)	0 (0.0)
Early serum creatinine	0	0	0 (0.0)	0 (0.0)	0 (0.0)
Serum albumin	36	4	40 (6.5)	22 (6.7)	18 (6.3)
Platelet count	0	0	0 (0.0)	0 (0.0)	0 (0.0)
Neutrophil count	0	0	0 (0.0)	0 (0.0)	0 (0.0)
Lymphocyte count	5	2	7 (1.1)	4 (1.2)	3 (1.0)
Monocyte count	4	1	5 (0.8)	3 (0.9)	2 (0.7)
Triglycerides	62	8	70 (11.4)	38 (11.6)	32 (11.2)
Admission glucose	25	5	30 (4.9)	17 (5.2)	13 (4.5)
Historical reference creatinine	8	0	8 (1.3)	4 (1.2)	4 (1.4)
Pre-index urine-output documentation	7	2	9 (1.5)	5 (1.5)	4 (1.4)
APACHE II score	8	2	10 (1.6)	6 (1.8)	4 (1.4)
SOFA score	9	3	12 (2.0)	7 (2.1)	5 (1.7)
Post-index AKI ascertainment	5	3	8 (1.3)	5 (1.5)	3 (1.0)
Post-index RRT initiation ascertainment	3	2	5 (0.8)	3 (0.9)	2 (0.7)
Twenty-eight-day mortality ascertainment	5	0	5 (0.8)	2 (0.6)	3 (1.0)
SII calculation	—	—	7 (1.1)	4 (1.2)	3 (1.0)
PIV calculation	—	—	10 (1.6)	6 (1.8)	4 (1.4)
TyG calculation	—	—	80 (13.0)	44 (13.4)	36 (12.6)
RIN-IM Score calculation	—	—	105 (17.1)	58 (17.6)	47 (16.4)
Any unavailable information leading to primary-analysis exclusion	—	—	135 (22.0)	74 (22.5)	61 (21.3)

B. Mutually exclusive patterns affecting primary-analysis eligibility

Pattern of unavailable core information	Patients, n (%) (N = 135)
Triglycerides only	35 (25.9)
Albumin only	15 (11.1)
Triglycerides and admission glucose	20 (14.8)
Albumin and triglycerides	15 (11.1)
Lymphocyte and/or monocyte counts	10 (7.4)
Albumin and admission glucose	10 (7.4)
Historical reference creatinine and/or pre-index urine-output documentation	12 (8.9)
Post-index AKI ascertainment	8 (5.9)
Post-index RRT initiation ascertainment	5 (3.7)
Twenty-eight-day mortality ascertainment	5 (3.7)
**Total**	**135 (100.0)**

AKI, acute kidney injury; APACHE II, Acute Physiology and Chronic Health Evaluation II; AUC, area under the receiver operating characteristic curve; CI, confidence interval; ICU, intensive care unit; MI, multiple imputation; OR, odds ratio; PIV, pan-immune-inflammation value; RIN-IM, Renal–Inflammatory–Nutritional–Metabolic Index; RRT, renal replacement therapy; SII, systemic immune-inflammation index; SMD, standardized mean difference; SOFA, Sequential Organ Failure Assessment; TyG, triglyceride–glucose index.

Supplementary Table S3. Comparison of patients included in the primary analysis and those excluded because of insufficient data.

A. Demographic and clinical characteristics

**Characteristic**	**Included patients (n = 480)**	**Excluded patients (n = 135)**	**Absolute SMD**
Age, years	64.7 ± 14.2	66.9 ± 14.7	0.154
Male sex, n (%)	286 (59.6)	80 (59.3)	0.007
Study center 1, n (%)	255 (53.1)	74 (54.8)	0.034
Hypertension, n (%)	258 (53.8)	78 (57.8)	0.081
Diabetes mellitus, n (%)	191 (39.8)	61 (45.2)	0.109
Chronic kidney disease, n (%)	90 (18.8)	31 (23.0)	0.104
Heart failure, n (%)	119 (24.8)	39 (28.9)	0.093
Chronic lung disease, n (%)	87 (18.1)	29 (21.5)	0.084
Active malignancy, n (%)	48 (10.0)	17 (12.6)	0.082
Sepsis at ICU admission, n (%)	216 (45.0)	70 (51.9)	0.137
APACHE II score	19.7 ± 7.5	21.3 ± 7.8 (n = 125)	0.210
SOFA score	6.2 ± 3.0	6.8 ± 3.1 (n = 123)	0.189

B. Outcomes among patients with ascertainable status

**Outcome**	**Included patients, n/N (%)**	**Excluded patients, n/N (%)**	**Absolute SMD**
Post-index AKI	189/480 (39.4)	53/115 (46.1)	0.136
Post-index RRT initiation	72/480 (15.0)	24/118 (20.3)	0.140
Twenty-eight-day all-cause mortality	157/480 (32.7)	54/130 (41.5)	0.184

Data are presented as mean ± standard deviation or n (%), unless otherwise specified. AKI, acute kidney injury; APACHE II, Acute Physiology and Chronic Health Evaluation II; AUC, area under the receiver operating characteristic curve; CI, confidence interval; ICU, intensive care unit; MI, multiple imputation; OR, odds ratio; PIV, pan-immune-inflammation value; RIN-IM, Renal–Inflammatory–Nutritional–Metabolic Index; RRT, renal replacement therapy; SII, systemic immune-inflammation index; SMD, standardized mean difference; SOFA, Sequential Organ Failure Assessment; TyG, triglyceride–glucose index.

Supplementary Table S4. Missing-data sensitivity analyses and center-specific discrimination for renal outcomes.

**A. Primary complete-case and multiple-imputation analyses**					
**Outcome**	**Analysis**	**Patients, n**	**Events, n (%)**	**Adjusted OR per RIN-IM point (95% CI)**	**p-value**
AKI	Primary complete-case analysis	480	189 (39.4)	1.31 (1.16–1.48)	<0.001
AKI	Multiple-imputation sensitivity analysis	595	242 (40.7)	1.29 (1.16–1.44)	<0.001
RRT initiation	Primary complete-case analysis	480	72 (15.0)	1.47 (1.25–1.73)	<0.001
RRT initiation	Multiple-imputation sensitivity analysis	598	96 (16.1)	1.44 (1.25–1.66)	<0.001

**B. Distribution of patients and renal outcomes by center**			
**Study center**	**Patients, n**	**AKI, n (%)**	**RRT initiation, n (%)**
Center 1	255	98 (38.4)	36 (14.1)
Center 2	225	91 (40.4)	36 (16.0)
Overall	480	189 (39.4)	72 (15.0)

**C. Center-specific discrimination**			
**Study center**	**Score**	**AUC for AKI (95% CI)**	**AUC for RRT initiation (95% CI)**
Center 1	RIN-IM	0.868 (0.821–0.915)	0.910 (0.859–0.961)
Center 1	APACHE II	0.789 (0.732–0.846)	0.811 (0.737–0.885)
Center 1	SOFA	0.813 (0.758–0.868)	0.837 (0.769–0.905)
Center 2	RIN-IM	0.854 (0.801–0.907)	0.893 (0.832–0.954)
Center 2	APACHE II	0.771 (0.706–0.836)	0.790 (0.705–0.875)
Center 2	SOFA	0.800 (0.738–0.862)	0.817 (0.738–0.896)

AKI, acute kidney injury; APACHE II, Acute Physiology and Chronic Health Evaluation II; AUC, area under the receiver operating characteristic curve; CI, confidence interval; ICU, intensive care unit; MI, multiple imputation; OR, odds ratio; PIV, pan-immune-inflammation value; RIN-IM, Renal–Inflammatory–Nutritional–Metabolic Index; RRT, renal replacement therapy; SII, systemic immune-inflammation index; SMD, standardized mean difference; SOFA, Sequential Organ Failure Assessment; TyG, triglyceride–glucose index.

Supplementary Table S5. Exploratory calibration and prediction-error measures for outcome-specific score-based models.

**A. Analysis population and reference prediction error**			
**Outcome**	**Patients, n**	**Events, n (%)**	**Constant-risk reference Brier score**
Incident AKI	480	189 (39.4)	0.2387
RRT initiation	480	72 (15.0)	0.1275

**B. The calibration and prediction-error estimates**				
**Outcome**	**Score-based model**	**Calibration-in-the-large (95% CI)**	**Calibration slope (95% CI)**	**Brier score (95% CI)**
Incident AKI	RIN-IM	0.01 (−0.22 to 0.24)	0.96 (0.78–1.14)	0.151 (0.133–0.169)
Incident AKI	APACHE II	−0.02 (−0.24 to 0.20)	0.97 (0.72–1.22)	0.184 (0.165–0.203)
Incident AKI	SOFA	0.00 (−0.22 to 0.22)	0.95 (0.73–1.17)	0.174 (0.155–0.193)
RRT initiation	RIN-IM	0.02 (−0.31 to 0.35)	0.92 (0.66–1.18)	0.080 (0.064–0.096)
RRT initiation	APACHE II	−0.03 (−0.33 to 0.27)	0.94 (0.57–1.31)	0.107 (0.089–0.125)
RRT initiation	SOFA	0.01 (−0.30 to 0.32)	0.93 (0.59–1.27)	0.100 (0.083–0.117)

The constant-risk reference Brier score was calculated as $\pi(1 - \pi)$, where π is the observed event proportion. AKI, acute kidney injury; APACHE II, Acute Physiology and Chronic Health Evaluation II; AUC, area under the receiver operating characteristic curve; CI, confidence interval; RIN-IM, Renal–Inflammatory–Nutritional–Metabolic Index; RRT, renal replacement therapy; SOFA, Sequential Organ Failure Assessment.
